# Assessment and Mitigation of Cardiovascular Risk for Prostate Cancer Patients: A Review of the Evidence

**DOI:** 10.1155/2022/2976811

**Published:** 2022-05-17

**Authors:** Patrick Davey, Kyriacos Alexandrou

**Affiliations:** ^**1**^ Northampton General Hospital, Cliftonville, Northampton NN1 5BD, UK; ^2^Ysbyty Gwynedd Hospital, Bangor, Penrhosgarnedd LL57 2PW, UK

## Abstract

**Background:**

Cardiovascular disease (CVD) is a common comorbidity in patients with prostate cancer. In this review, we summarize the published literature on the association of cardiovascular risk with androgen deprivation therapy (ADT) treatment and explore the potential differences between the gonadotropin-releasing hormone (GnRH) agonists and antagonists and the molecular mechanisms that may be involved. We also provide a practical outlook on the identification of underlying CV risk and explore the different stratification tools available.

**Results:**

While not definitive, the current evidence suggests that GnRH antagonists may be associated with lower rates of certain CV events vs agonists, particularly in patients with preexisting CVD. Risk reduction strategies such as lifestyle advice, consideration of ADT modality, and comedications may help to reduce CV risk factors and improve outcomes in prostate cancer patients receiving ADT.

**Conclusions:**

Given all the data that is currently available, identification of baseline CV risk factors may be key to risk mitigation in patients with prostate cancer receiving ADT.

## 1. Introduction

Cardiovascular disease (CVD) is a common concomitant condition in patients with prostate cancer and according to the European Association of Urology guidelines [1], CV mortality now exceeds prostate cancer as the most common cause of death. Several studies have shown that the risk of CVD is higher in prostate cancer patients compared with the general population [2, 3].

As a result, the cardiovascular effects of prostate cancer therapies have been investigated. Androgen deprivation therapy (ADT), while an effective treatment for advanced hormone-dependent prostate cancer, has been associated with a number of cardiometabolic side effects including decreased insulin sensitivity, changes in lipid profile, and an increased risk of thromboembolic and cerebrovascular events [4, 5]. Currently, available pharmaceutical ADTs include luteinizing hormone-releasing hormone (LHRH) agonists, such as leuprorelin, goserelin, triptorelin, and buserelin acetate, and the gonadotrophin-releasing hormone (GnRH) antagonist such as degarelix. For semantic simplicity, we will now refer to LHRH agonists as GnRH agonists, in contrast to GnRH antagonists.

It is GnRH agonist treatment that has specifically been associated with increased CV morbidity and mortality in several observational studies [2, 6–8]. Among them, a population-based study using data from the Surveillance, Epidemiology and End Results (SEER)-Medicare linked database demonstrated an increased risk of coronary heart disease, myocardial infarction (MI), and sudden cardiac death [7, 9] with GnRH agonist use, but not with bilateral orchiectomy. While both treatments may increase CV risk due to the lack of testosterone, GnRH agonists further increase the risk of thromboembolic events. Subsequently, the FDA has required manufacturers of GnRH agonists to include a warning of the increased risk of diabetes, heart attack, and sudden cardiac death [10].

This review will summarize recent evidence on the cardiovascular risks associated with GnRH agonists and antagonists and provide advice on the assessment and mitigation of cardiovascular risk for prostate cancer patients in clinical practice.

## 2. Methodology

This review was conducted through a comprehensive search in PubMed and ClinicalTrials.gov with the following key terms: “prostate cancer” AND “agonists” AND “antagonist” AND “cardiovascular” according to the recommendations in the PRISMA (Preferred Reporting Items for Systematic Review and Meta-analyses) statement. The initial search led to 106 publications—review articles, case reports, and editorials were removed, with priority given to relevant clinical trials, meta-analyses, and real-world evidence observational studies published in the last 10 years. In total, 32 articles were retrieved, and once duplicates were removed, 29 articles were reviewed as a result of the selection process (Supplementary Figure 1). Due to the small number of articles retrieved from the search, no minimum sample size was defined. The Downs and Black checklist was used for assessing the risk of bias in the individual studies reported [11]. “A preprint of this article has previously been published [12].

### 2.1. Randomized Controlled Trials (RCTs)

Large prospective RCTs in patients with prostate cancer on ADT with major adverse cardiovascular events as primary or secondary endpoints are currently lacking (Table 1) [15]. The PRONOUNCE (NCT02663908) study is an RCT comparing the CV safety of degarelix with leuprolide in prostate cancer patients with predefined cardiovascular disease [16, 17]. The study was originally planned to complete in October 2021. The primary endpoint was time from randomization to the first confirmed, adjudicated occurrence of a MACE, which is defined as a composite of all-cause death, nonfatal myocardial infarction, or nonfatal stroke through 12 months of ADT treatment. Due to the slower than projected enrolment and fewer than projected primary outcome events, enrolment was stopped early and the study continued as per protocol with the subjects already included. 544 patients were randomized and dosed vs. 900 initially targeted (60%). 26 subjects had a MACE, 15 in the degarelix group and 11 in the leuprolide group (*p*=0.53) [17]. Because of the lower than anticipated number of events (26 vs. 41 expected for 544 subjects), the study did not generate any statistically significant results. The lower than expected event rate (in both arms) is most likely due to a high number of CV interventions (97% were on CV medications-lipid modifying agents, agents acting on the renin-angiotensin system or beta-blockers) and that all patients in the study were under the close care and monitoring of a cardiologist. However, the study does provide a model for the interdisciplinary collaboration between urologists, oncologists, and cardiologists with a shared goal of evaluating the impact of cancer therapies on cardiovascular outcomes [17].

Cardiovascular morbidity among patients with prostate cancer and preexisting CV disease receiving antagonists and agonists has been investigated in a smaller randomized controlled study (*N* = 80) [13]. The aim of this study was to compare endothelial function and the number of major adverse cardiovascular and cerebrovascular events (MACCEs) defined as death, myocardial infarction, cerebrovascular event, heart catheterization, and stent insertion. Endothelial function, measured by an EndoPAT device, did not differ between the treatment groups at 12 months (mean ± SD RHI 2.07 ± 0.15 vs 1.92 ± 0.11, *p*=0.42). This is likely due to the high proportion of patients with severe endothelial dysfunction at baseline. After 12 months, 8 (20%) patients randomized to GnRH agonist had a MACCE compared to 1 (3%) treated with the antagonist (ARR = 18.1% (95% CI 4.6–31.2), NNT = 6) [13]. High levels of N-terminal pro-B-type natriuretic peptide (NT-pro-BNP) and high-sensitivity troponin (hsTn) at baseline were associated with the development of new CV events in the agonist group but not in the antagonist group [18]. This study is limited due to the small sample size and the suitability of the primary endpoint which was not met. Further, a large proportion of patients in this study had localized prostate cancer which may not be representative of the licensed indication in some countries where it is used in a more advanced setting.

Recently, a phase III, randomized controlled trial compared the GnRH oral antagonist, relugolix (*N* = 622), with the GnRH agonist, leuprorelin (*N* = 308). At baseline, more than 90% of all patients had at least one CV risk factor including lifestyle risk factors, diabetes, hypertension, and a history of a major adverse cardiovascular event (MACE). 13.5% had a history of a MACE in the relugolix group vs 14.6% in the leuprolide group. Although not included as a primary or secondary endpoint, a prespecified safety analysis showed the incidence of a MACE was 3.6% in the relugolix group and 17.8% in the leuprolide group (ARR = 14.2, NNT = 7) [14].

There is currently a lack of hypothesis-driven randomized controlled trials in patients with baseline CV disease receiving GnRH agonists and antagonists that have major adverse cardiovascular or cerebrovascular events as primary endpoints. The inconclusive results from the PRONOUNCE study highlight the difficulty of using RCTs to evaluate the efficacy and safety of cancer therapies. They are expensive, face recruitment and retention difficulties and take a long time to complete [19].

### 2.2. Meta-Analyses of Randomized Controlled Trials

Due to the lack of randomized controlled data investigating the effects of androgen deprivation therapy on major adverse cardiovascular events as primary or secondary endpoints, a number of meta-analyses analyzing adverse event data from existing RCTs have been carried out (Table 2).

Data pooled from 5 prospective, randomized trials (*n* = 1925) comparing the GnRH antagonist degarelix and GnRH agonists showed patients in the degarelix group had a lower risk for death after adjusting for baseline factors [24]. This difference in overall survival was unlikely to have been driven by prostate cancer deaths (only four patients died as a result of disease progression), but by cardiovascular events, with 51% of the deaths occurring in the 29% of patients with preexisting cardiovascular disease [24]. This hypothesis was first investigated by analyzing the data from 6 prospective, phase 3 randomized controlled trials (*N* = 2328) comparing degarelix (*N* = 1491) with the agonists (either leuprolide, *n* = 379; or goserelin, *n* = 458) [20]. Approximately 30% of patients in both treatment groups had preexisting CV disease. In the total patient population, there was a 40% relative risk reduction of a CV event or death in patients treated with degarelix compared with patients treated with a GnRH agonist (HR: 0.60; 95% CI, 0.41–0.87; *p*=0.008). No differences were observed in the incidence of either death or CV events among the men who had no baseline CV disease. However, in those patients with preexisting CV disease (*N* = 708), there were significantly fewer cardiac events or deaths experienced by patients receiving a GnRH antagonist (6.5%) compared with patients receiving GnRH agonists (14.7%) (ARR = 8.2%, NNT = 12) [20]. It is important to note that the RCTs analyzed in this study were not blinded to treatment allocation and there could be the possibility of underreporting of CV events in the GnRH antagonist group. Further, none of these studies had CV events as a primary or secondary endpoint and were reported as adverse events which were not systematically validated.

Since this initial meta-analysis in 2014, several other studies have been published. Abufaraj et al. carried out a meta-analysis of 8 clinical trials in 20 published studies that showed GnRH antagonists were associated with fewer cardiovascular events than GnRH agonists (RR:0.52, 95% CI 0.34–0.80) [21]. The risk of bias in this study was low due to the inclusion of only RCTs; however, the meta-analysis is limited due to the short follow-up periods. Sciarra et al. reported a similar outcome with an analysis of 3 clinical trials showing treatment-related severe CV side effects including prolonged QT interval, angina pectoris, atrial fibrillation, cardiac failure, and MI) reported in 1.6% of patients on degarelix and 3.6% of patients on GnRH agonists (OR−0.55, 95% CI; 0.26–1.14,*P* > 0.1) [22]. One recent meta-analysis showed conflicting results with the incidence of CVD with GnRH agonists and antagonists equal (RR: 0.98, 95% CI 0.94–1.02) [23]. However, the analysis in this study is not robust with a meta-analysis classed as an RCT leading to duplicate analyses and results should be interpreted with caution.

The majority of results from the meta-analyses demonstrate a recurring pattern by which ADT increases the risk of CV events, which appears to be driven by GnRH agonists [25]. The meta-analyses reported here all have similar limitations due to the nature of the RCTs included in their analysis including, the lack of CV primary and secondary endpoints, short follow-up times, and lack of blinding study subjects. Further, it is important to note that there is an overlap, with most of these studies analyzing the same clinical trials—notably CS28, CS30, CS31, CS21, and CS35.

### 2.3. Real-World Observational Studies

The lack of randomized controlled trial data has led to an increasing interest in real-world data (RWD) as an alternative way to evaluate the safety of prostate cancer therapies in clinical practice (Table 3). Analysis of prostate cancer patients from a UK primary care database (*N* = 9,081) showed that patients receiving degarelix had a higher baseline PSA than those initiating therapy with a GnRH agonist. In addition, more patients receiving degarelix had preexisting CVD compared with patients receiving GnRH agonists. However, despite this difference in preexisting CVD, in a post hoc analysis, the relative risk of experiencing any cardiac event was lower with degarelix than with all GnRH agonists (6.9% vs 17.7%; 0.39 [0.191, 0.799]; *p*=0.01) [26]. The results of this study are limited due to the low number of patients on the GnRH antagonist. Further, data from secondary care has not been analyzed which may result in bias due to patient selection and missing event reporting.

Similar data was shown from Italian pharmacy and hospital databases. The incidence rate of CV events in the agonist group was 8.80/100 person-years vs 6.24/100 person-years in the antagonist group (*P*=0.002). The time to an event was beyond one year in both groups (mean years ± SD; agonist 1.6 ± 1.3 vs antagonist 1.2 ± 1.1) [27]. Lower cardiac events have also been reported from analysis of a pharmacovigilance database (Vigibase) that collates adverse event reporting data from more than 130 countries [28]. Using disproportionality analysis, the authors found GnRH antagonists had no signal for any cardiac event other than heart failure (Reporting odds ratio (ROR) 1.91). The GnRH agonists showed a significant incidence of cardiac events (ROR 1.20) which was driven by MI (ROR 1.76) and heart failure (ROR 2.02). The time from initiation of therapy to CV event was over 1-year (mean 541.9 days, SD 909.6) [28]. A similar study was carried out using the FDA Adverse Event Reporting System (FAERS) database [29]. Of 20 million CV events, 50, 000 were related to prostate cancer therapies. GnRH antagonists were associated with fewer CV event reports (mainly arterial vascular events, venous thromboembolism, and arrhythmias) than GnRH agonists (Reporting Odds Ratio ROR = 0.70 [95% CI 0.59–0.84], *p* < 0.001). Further, a combination of GnRH agonists with any of the first- or second-generation androgen receptor inhibitors or abiraterone showed a 35% increase in the odds of a CV event compared to when they were used in combination with a GnRH antagonist ((arterial vascular events, e.g., MI/CAD/hypertension and arrhythmias) (ROR = 0.64 [0.54–0.81], *p*=0.0003)) [29].

Such observational studies are always limited by the risk of confounding bias. They often have key clinical data missing due to differences in data capture and reporting which are not always accounted for in the statistical analysis and study results are vulnerable to confounding in terms of patient selection. Further, the studies which have used pharmacovigilance adverse event data are always subject to reporting bias.

In contrast, other real-world data studies have not demonstrated a clear CV risk benefit with the GnRH antagonist (Table 3). A French study analyzed data from a health insurance database (35, 118 ADT users) which showed no significant association between GnRH antagonists and CV risk (adjusted HR [95% confidence interval [CI] 1.2 [0.7–2.1]]) [30]. Combined androgen blockade was associated with an increased CV risk (adjusted HR [95% confidence interval [CI] 1.6 [1.3–2.1]]), and antiandrogen therapy was associated with a decreased risk of ischemic events (adjusted HR [95% confidence interval [CI] 0.6 [0.4–0.9]]) compared with GnRH agonist therapy [30].

A retrospective analysis from a German claims database showed a higher proportion of patients treated with the antagonists had metastases compared to the agonist (38% vs 30%, *P*=0.002). In these patients, there were no significant differences in the incidence of CVD or diabetes between GnRH agonists or antagonists overall, although there was a significant increase in hypertension in patients receiving a GnRH agonist compared with those receiving a GnRH antagonist (16.4% vs 6.9%, *P*=0.002) [31]. The data analyzed from this study is health insurance data which relies upon accurate coding—this can often lead to the underrepresentation of certain events or can underestimate the prevalence of a condition. Further, it is not possible to identify other comorbidities and lifestyle factors that may impact the results.

A recent registry study by Cardwell et al. in a cohort of >22,000 men with prostate cancer from the Scottish Cancer Registry who were prescribed ADTs between 2009 and 2015 found a 30% increase in CV risk with ADT compared with untreated patients; they reported a 30% increase in risk with GnRH agonists and a 50% increase in risk with degarelix [32]. The authors state the association of higher CV risk with ADT compared with untreated patients was largely driven by CV events in GnRH agonist-treated patients; this may not allow for a conclusion to be drawn regarding the increase of CV risk with degarelix treatment, particularly given a risk of a type 1 error (false positive) due to the small sample size.

Real-world data was analyzed from 5 different countries (United Kingdom, Scotland, Belgium, Netherlands, and France) to investigate CV risk in men with prostate cancer [33]. In total, 48,757 men were treated with GnRH agonists and 2,144 with antagonists. There was no difference in risk of any CVD (ischemic heart disease, acute myocardial infarction, arrhythmia, heart failure, and stroke) between GnRH antagonists and agonists (HR: 1.25; 95% CI: 0.96–1.61; I2: 64%). Men on GnRH antagonists showed an increased risk of acute myocardial infarction (HR: 1.62; 95% CI: 1.11–2.35; I2: 0%) and arrhythmia (HR: 1.55; 95% CI: 1.11–2.15, I2: 17%) compared to GnRH agonists. The authors state that in such a large prospective cohort study it is difficult to fully homogenize study variables due to the varied data sources used. For example, the UK had no records for AMI because the data source used (THIN) was from a primary care setting. This heterogeneity in data sources may have affected the results of the study [33]. The databases used in this study overlap with the previous studies reported. For example, the Scottish Cancer Registry database was used in the Cardwell study [32] and the French Health National Database (SNIIRAM) was analyzed in the Scailteux study above [30].

Real-world data observational studies provide a powerful insight into patient outcomes with much longer follow-up times compared to RCTs. However, they are limited by bias/confounding, differing sample sizes, missing data, and the lack of randomization and blinding.

In summary, the available data indicate that GnRH agonists increase CV events and CVD and that the increase appears to be greatest in patients with preexisting CVD. While not definitive, some of the current evidence suggests that GnRH antagonists may be associated with lower rates of certain CV events vs agonists, particularly in patients with preexisting CVD.

We have reached this conclusion as the highest-grade evidence from randomized clinical trials comparing GnRH agonists with antagonists is uniform in finding lower CV event rates with GnRH antagonists. Furthermore, although heterogeneous, the data from real-world observational studies does not seem to negate this view.

### 2.4. Potential Mechanisms Underlying a Differential CV Effect for GnRH Agonists and Antagonists

**Figure 1:**
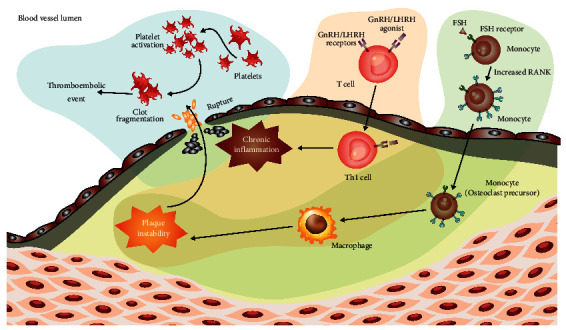
Proposed mechanism of differing CV risk between antagonists and agonists.

There are two potential mechanisms speculated to underlie the difference in CV risk between GnRH antagonists and agonists. The first centers on differential effects of the two agents on T lymphocyte activation and destabilization of atherosclerotic plaques, and the second focuses on the role of FSH (Figure 1) (Table 4) [4, 38].

GnRH receptors are expressed are on T lymphocytes which are stimulated by GnRH agonists [36]. This triggers an inflammatory cascade, stimulating the proliferation of T cells and the production of proinflammatory cytokines. Activated macrophages produce matrix metalloproteinases (MMPs) which degrade the fibrotic cap within vulnerable plaques [20, 36, 37, 39, 40]. This increases the risk of atherosclerotic plaque rupture [38], and disruption of atheromatous plaques is a frequent cause of fatal coronary thrombi [41]. Unlike GnRH agonists, GnRH antagonists lack the ability to activate T lymphocytes, thus maintaining plaque stability (Figure 1) [38]. Currently, research on this mechanism remains in preclinical studies. In ApoE(−/−) mice, treatment with the GnRH agonist leuprolide, but not the GnRH receptor antagonist degarelix, induced atherosclerotic plaque instability [35].

A parallel hypothesis centers around the role of FSH. Animal studies have implicated FSH in cardiovascular morbidity with mouse models showing dysfunctional fat increased in an FSH-dependent manner (Table 4) [34]. Further, antagonist-treated mice had significantly lower levels of FSH and a lower percentage of adipose tissue and had half the number of atherosclerotic plaques and less inflammation compared with agonist or orchiectomy [34]. FSH has also been shown to stimulate proinflammatory cytokine release from immune cells, providing another mechanism by which plaque stability may be disrupted (Figure 1) [4]. Clinical data has reflected some of these findings with clinical trial data showing FSH is suppressed to a greater extent with antagonists compared to agonists. Additionally, in a prospective study, patients with less than a 60% decrease in FSH levels during the ﬁrst three months of treatment had a higher risk of developing a cardiovascular event (40% vs 10%, *p*=0.005) [42]. Further clinical research is needed to explore these mechanisms and to determine whether preclinical findings and hypotheses translate into differences in risk in a clinical setting.

## 3. Androgen Deprivation Therapy and Diabetes

Given the association of type 2 diabetes mellitus with CVD, CV morbidity, and mortality [43], data assessing the potential impact of ADT on the development of diabetes are also of interest in this context. A meta-analysis of eight studies assessing diabetes-related outcomes in patients with prostate cancer who received ADT compared with ADT-naïve patients showed that the pooled incidence of diabetes was 39% higher in patients receiving ADT compared with control groups (risk ratio [RR] 1.39, 95% confidence interval [CI]; 1.27–1.53; *p* < 0.001). In subgroup analyses, diabetes was found to be associated with GnRH agonists (RR 1.45, 95% CI 1.36–1.54; *p* < 0.001), GnRH agonists + antiandrogens (RR 1.40, 95% CI 1.01–1.93; *p*=0.04), and orchiectomy (RR 1.34, 95% CI 1.20–1.50; *p* < 0.001), but not with antiandrogens alone (RR 1.33, 95% CI 0.75–2.36; *p*=0.33). Diabetes risk appeared to increase with the length of ADT treatment (≤6 months: RR 1.29, 95% CI 1.12–1.49, *p*=0.0004; >6 months: RR 1.43, 95% CI 1.22–1.68, *p* < 0.001) [44].

### 3.1. Assessment and Mitigation of CV Risk in Clinical Practice

A central component of any risk reduction strategy is education to increase awareness among prostate cancer patients of the signs and symptoms of CVD and modifiable CV risk factors. In the absence of formal guidelines for the prevention and management of CVD in prostate cancer patients receiving ADT, strategies can be put in place to control for CV risk factors in these patients—the ABCDE paradigm (Awareness and Aspirin, Blood Pressure, Cholesterol and Cigarette, Diet and Diabetes, and Exercise) developed by Bhatia and colleagues is one example [45].

The INTERHEART study showed that nine modifiable risk factors were significantly associated with acute MI in both men and women and taken together these factors accounted for more than 90% of the population-attributable risk: hypertension, diabetes, physical activity, alcohol use, abnormal lipids, smoking, abdominal obesity, unhealthy diet, and psychosocial stress [46]. This study confirms the importance of multiple risk factor intervention to reduce the overall risk of cardiovascular events.

The group at the highest risk of CV events are those who have already had a vascular event, followed by those with the largest number of risk factors (age, diabetes, and smoking) [45]. The risk of those without preexisting CVD and risk factors can be formally calculated by QRISK. Thus, screening for known and undiagnosed metabolic and CV risk factors and risk calculation QRISK or JBS3 is key to risk mitigation, enabling stratification of prostate cancer patients according to the level of risk (e. g. “CV history/high CV risk” vs “no history/low risk”) [47]. The use of such tools is recommended in a recent publication by the International Cardio-Oncology Society for patients receiving ADT [48]. The Canadian Urological Association has recently published guidelines on the screening and management of CV health in prostate cancer patients with simple “STAMP” questions (Stroke, Transient Ischemic attack, Abdominal Aortic Aneurysm, Myocardial Infarction, Peripheral arterial disease) and the recommendation for every patient to collect routine medical history; perform a physical examination; determine the lipid profile, measure HbA1c, uric acid, serum electrolytes, and creatinine; and perform complete blood count (CBC) and electrocardiogram (ECG) [49, 50].

Ongoing monitoring and treatment of CV risk factors are important. For example, treatment with GnRH agonists can lead to diabetes (due to the development of insulin resistance or altered insulin sensitivity), obesity, and other metabolic changes, including changes in lipid levels, which can exacerbate atherosclerotic diseases [44, 51–55].

In addition, key changes in patients' habits should be encouraged, the goal being to maintain a healthy lifestyle. Advice should be given regarding smoking cessation, healthy diet, weight control, and daily exercise [45]. However, patients with CV symptoms (which should be asked for) may also need targeted investigation and treatment by a cardiologist.

**Figure 2:**
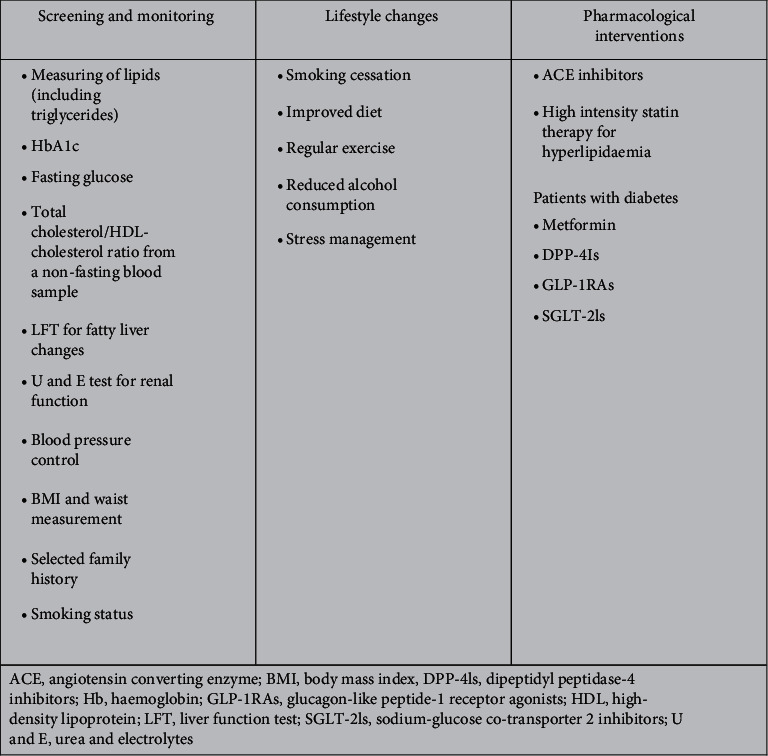
Box 1 key CV risk screening and monitoring strategies and lifestyle and pharmacological interventions.

**Figure 3:**
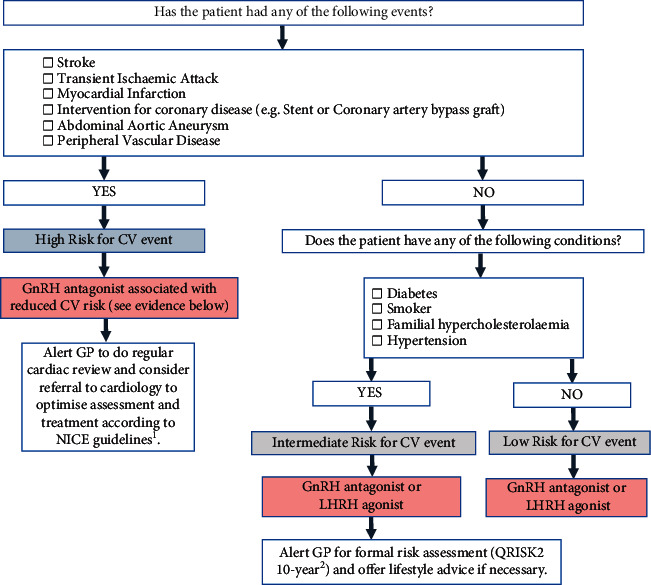
Cardiovascular risk assessment tool.

Pharmacological interventions include angiotensin-converting enzyme inhibitors, which are the recommended first-choice agents for hypertension due to mortality-risk reduction in patients with diabetes and CVD, and possibly improved outcomes in cancer patients, including those with prostate cancer [45, 56]. Hyperlipidemia should be treated with high-intensity statin therapy, especially in the presence of diabetes or CVD (Figure 2) [45]. While metformin has been the preferred agent for the treatment of diabetes in this population due to its favorable effects on the metabolic syndrome [45], newer diabetes treatments include dipeptidyl peptidase-4 inhibitors (DPP-4Is), glucagon-like peptide-1 receptor agonists (GLP-1RAs), and sodium-glucose cotransporter 2 inhibitors (SGLT-2Is; Figure 3). In large cardiovascular outcome trials, which have been a requirement by the US Food and Drug Administration since 2008, GLP-1RAs and SGLT-2Is have demonstrated CV benefits in addition to improving glycaemic control, and empagliflozin and canagliflozin (both SGLT-2Is) demonstrated significant reductions in major adverse cardiovascular events. Guidelines from the American Diabetes Association (ADA), the European Association for the Study of Diabetes (EASD), and the American College of Cardiology recommend SGLT2Is or GLP-1RAs in patients with type 2 diabetes and additional risk factors for CVD since these agents have been shown to improve cardiovascular outcomes over 2–5 years [57, 58]. The 2019 update of the 2018 ADA and EASD guidelines specifies that the decision to treat high-risk individuals with these agents should now be considered independently of baseline HbA1c or individualized HbA1c target [59]. On the other hand, DPP-4Is only showed low to moderate glycaemic efficacy and no CV or renal benefit, with some agents in this class having been associated with an increase in heart failure [60].

In prostate cancer patients, appropriate ADT should be undertaken, with particular caution in patients with preexisting CVD and/or >10% CVD 10-year risk (assessed using QRISK2), which is the treatment threshold for primary prevention of CVD as per NICE guidelines [61]. A checklist for urologists, oncologists, and clinical nurse specialists may prove useful, encompassing CV history, concomitant medication, lifestyle approach, ADT, risk of recurrence, and tumor aggressiveness. Figure 3 provides a template CV risk assessment tool, to help risk stratify patients and consider ADT modality accordingly.

## 4. Limitations

This literature review has summarized all the data available on CV events in prostate cancer patients receiving GnRH agonists and antagonists and provides practical guidance on how to identify and mitigate this risk. However, it is limited by the small number of publications available within the search criteria. As a result, publications were not excluded due to quality, low patient numbers, or robustness of study design. It is also important to note that due to the lack of RCTs with MACCE as primary or secondary endpoints, most of the data reviewed are from meta-analyses or retrospective observational studies. However, the strengths and limitations of each study have been explored and stated within this review (adapted from [62]).

## 5. Summary

This review of the literature indicates that prostate cancer patients on androgen deprivation therapy are at an increased risk of CV events. While not definitive, some of the current evidence suggests that GnRH antagonists may be associated with lower rates of certain CV events vs agonists, particularly in patients with preexisting CVD. Given all the data that is currently available, identification and assessment of CV risk factors may be key to risk mitigation in patients with prostate cancer receiving ADT. In addition, appropriate lifestyle and other pharmacological interventions should be recommended, and CV risk factors should be monitored long-term.

## Figures and Tables

**Table 1:** ADT and cardiovascular outcomes from randomized controlled trials.

Authors	Patient setting	Country	Patient numbers and study endpoints	Follow-up time	CV outcomes
PRONOUNCE study (NCT02663908)	Pharmaceutical sponsored phase III interventional study	Canada, Europe, South Africa, United States	*N* = 545 Results not yet published.	1 year	Results not yet published.
Margel et al. *J* Urol 2020 [13]	Investigator led pharmaceutical sponsored phase II interventional study	Israel	*N* = 80 The study primary end point was to compare endothelial function between the 2 arms. Cardiovascular events were a predefined secondary outcome including death, MI, CVA, a transient ischemic attack, heart catheterization with or without intervention and cardiac related hospitalization. MACCEs were defined as death, MI, CVA, and heart catheterization with stent insertion.	1 year	After 12 months, 8 (20%) patients randomized to GnRH agonist had a MACCE compared to 1 (3%) treated with the antagonist. The absolute risk reduction of a CV event or death was 18.1% (95% CI 4.6–31.2), NNT = 6.
Shore et al. NEJM 2020 [14]	Pharmaceutical sponsored phase III interventional study	North America and Japan	*N* = 930 The primary endpoint was the sustained castration rate, defined as the cumulative probability of testosterone suppression to less than 50 ng per deciliter during receipt of trial treatment from day 29 through 48 weeks. Cardiovascular or cerebrovascular risk factors included prespecified event terms in the MACE query and a manual search of known risk factors, including hypertension; dyslipidemia; diabetes; a history of myocardial infarction or cardiovascular disease; a history of stroke, transient ischemic attack, or cerebral hemorrhage; peripheral arterial disease; atrial fibrillation and other arrhythmias; heart-valve disease; chronic obstructive pulmonary disease; chronic kidney disease; chronic liver disease; carotid-artery stenosis or occlusion; venous thromboembolic events; and heart failure.	Median follow-up time 52 weeks	In a prespecified analysis, in men with a history of MACE, the incidence of major adverse cardiovascular events (MACE) was lower in the relugolix group than in the leuprolide group (3.6% vs. 17.8%, respectively). ARR = 14.2, NNT = 7.

**Table 2:** ADT and cardiovascular outcomes from meta-analyses of randomized controlled trials.

Authors	Patient setting	Country	Patient numbers and study endpoints	Follow-up time	CV outcomes
Albertsen et al. Eur Urol 2014 [20]	Meta-analysis of 6 phase III RCTs	United Kingdom, USA, Western Europe, Scandinavia, global	*N* = 2328 The number of deaths from any cause and the number of cardiac events among all men receiving any form of ADT. Cardiac events included; arterial embolic and thrombotic events, hemorrhagic or ischemic cerebrovascular conditions, myocardial infarction, or other ischemic heart diseases.	3–14 months	In patients with preexisting CV disease at baseline, there was a 56% relative risk reduction of a CV event or death in patients treated with degarelix compared with patients treated with a GnRH agonist (HR: 0.44; 95% CI, 0.26–0.74; *p*=0.002). The absolute risk reduction of a CV event or death was 8.2%, NNT = 12.
Abufaraj et al. Eur urol [21]	Meta-analysis of 8 phase III RCTs	United Kingdom, USA, and Western Europe, Scandinavia, USA global, Japan, and Israel	*N* = 2,632 CV events; arterial embolic and thrombotic events, ischemic cerebrovascular events, myocardial infarction, and other ischemic heart diseases.	3–14 months	GnRH antagonist was associated with fewer cardiovascular events (RR: 0.52, 95% CI: 0.34–0.80).
Sciarra et al. Medicine [22]	Meta-analysis of 5 phase III RCTs	The United Kingdom, western Europe, Scandinavia, global	*N* = 1719 Treatment-related severe cardiovascular side effects; QT interval increase, angina pectoris, atrial fibrillation, cardiac failure, and myocardial ischemia.	3–14 months	Treatment-related severe cardiovascular side effects were reported in 1.6% and 3.6% of patients in the degarelix and GnRH agonists group, respectively (OR = 0.55, 95% CI: 0.26–1.14, *P* > 0.1).
Ma et al. Minerva Urologica *e* Nefrologica [23]	Meta-analysis of 4 RCTs and 2 observational studies	Canada, Germany, France, Sweden, and the Netherlands	*N* = 32,887 CVD outcomes (not specified).	4–14 months	The results of the meta-analysis showed that compared with the GnRH agonist, the incidents of CVD were equal to GnRH antagonist therapy for the patient with PCa (RR 0.98, 95% CI 0.94–1.02).

**Table 3:** ADT and cardiovascular outcomes from real-world observational studies.

Authors	Patient setting	Country	Patient numbers and study endpoints	Follow-up time	CV outcomes
Davey et al. W *J* Urol [26]	Retrospective population-based cohort study (RWE) from UK primary care database	United Kingdom	*N* = 9,081 The incidence of cardiac events following initiation of GnRH antagonist (degarelix) or GnRH agonist (leuprorelin, goserelin, or triptorelin) as therapy in patients with prostate cancer. CV events included; heart failure; myocardial infarction (MI); arrhythmia; ischemic heart disease.	≤7 years	The relative risk of experiencing any CV event was lower with degarelix than with all GnRH agonists (6.9% vs 17.7%) (ARR = 10.8, NNT = 9).
Perrone et al. Therapeutics and Clinical Risk Management [27]	Retrospective population-based cohort study (RWE) from beneficiaries database, pharmacy database, hospital database with primary and secondary data	Italy	*N* = 9,785 The incidence rate of CV events (acute myocardial infarction, ischemic heart diseases, cerebrovascular diseases, cardiac dysrhythmias, heart failure, atherosclerosis, aneurism, other CV-related conditions) was calculated among patients not switching to androgen deprivation therapy (ADT) in the overall cohort and in a subcohort of patients without previous CV events.	≤4 years	The incidence rate of CV events was significantly higher in patients treated with GnRH agonists rather than degarelix (8.80 vs 6.24 per 100 person-year, *p* value 0.002), with a mean time to CV event beyond 1 year.
Cone et al. BJU Int. [28]	Retrospective Pharmacovigilance database (Vigibase)	Worldwide	Cardiovascular reactions related to GnRH antagonist (degarelix) or agonist (leuprolide, goserelin, triptorelin, histrelin) therapy for prostate cancer. CV events included; myocardial infarction (MI), heart failure (HF), carditis (cardiomyopathies, pericarditis, and myocarditis), new valvular dysfunction, and new arrhythmias.	N/A	GnRH antagonists were associated with fewer CV event reports (mainly arterial vascular events, venous thromboembolism, and arrhythmias) than GnRH agonists (reporting odds ratio ROR = 0.70 [95% CI 0.59–0.84], *p* < 0.001).
Zhang et al. J. Urol [29]	Retrospective Pharmacovigilance database (FDA Adverse Event Reporting System)	USA	CV events included; arterial vascular events (coronary artery disease [CAD], myocardial infarction [MI], ischemic stroke, peripheral vascular disease [PVD], and hypertension requiring hospitalization), heart failure, venous thromboembolism (VTE; deep venous thrombosis and pulmonary embolism), and arrhythmias (atrial fibrillation and QT prolongation).	N/A	GnRH antagonists were associated with fewer CV event reports (mainly arterial vascular events, venous thromboembolism, and arrhythmias) than GnRH agonists (reporting odds ratio ROR = 0.70 [95% CI 0.59–0.84], *p* < 0.001).
Scailteux et al. Eur *J* cancer [30]	Retrospective population-based cohort study (RWE)—secondary care database	France	*N* = 35,118 Occurrence of “ischemic events”.	2-3 years	No significant association between GnRH antagonists and CV risk, although combined androgen blockade was associated with an increased risk, and antiandrogen therapy was associated with a decreased risk of ischemic events compared with GnRH agonist therapy.
Hupe et al. Front Oncol [31]	Retrospective population-based cohort study (RWE) using a health insurance claims database	Germany	*N* = 2,382 To analyze real-world information on healthcare characteristics and treatment patterns in patients with locally advanced or metastatic PCa dependent on the prescribed GnRH agonist/antagonist agents (GnRHa) in the first 3 years after initiation.	≥2 years	No significant differences in the incidence of CVD or diabetes between GnRH agonists or antagonists overall, although there was a significant increase in hypertension in patients receiving a GnRH agonist (16.4%) compared with those receiving a GnRH antagonist (6.9%, *p*=0.022)
Cardwell et al. Epidemiology [32]	Retrospective population-based cohort study (RWE) from the cancer registry, secondary care, community care, and death records	Scotland	*N* = 20,216 Cardiovascular events consist of either death from cardiovascular disease or hospitalization for cardiovascular disease. CV disease types included acute myocardial infarction, stroke, venous thromboembolism, heart failure, arrhythmia, and other cardiovascular diseases.	3 years	30% increase in CV risk with ADT compared with untreated patients; they report a 30% increase in risk with GnRH agonists and a 50% increase in risk with degarelix.
George et al. International Journal of Cancer [33]	Retrospective population-based cohort study (RWE) using data from primary care, cancer registry, secondary care, community care, insurance claims data, and death records	United Kingdom, Scotland, Belgium, Netherlands, and France	*N* = 50,909 To combine real-world data from 5 countries to compare the risk of CVD between agonists and antagonists. CVD included; ischemic heart disease (IHD), acute myocardial infarction (AMI), arrhythmia, heart failure (HF), and stroke.	≤7 years	There was no difference in risk of any CVD for men on GnRH antagonists and agonists (HR: 1.25; 95% CI: 0.96–1.61; I2: 64%). Men on GnRH antagonists showed an increased risk of acute myocardial infarction (HR: 1.62; 95% CI: 1.11–2.35; I2: 0%) and arrhythmia (HR: 1.55; 95% CI: 1.11–2.15, I2: 17%) compared to GnRH agonists.

**Table 4:** ADT and cardiovascular outcomes from preclinical and *in vitro* studies.

Authors	Animal model	Patient numbers and study endpoints/objectives	CV outcomes
Hopmans SN, et al. Urol Oncol [34]	Low-density lipoprotein receptor knockout mice (LDL KO)	*N* = 9–13/group To investigate the effects of bilateral orchiectomy, GnRH agonist, and GnRH antagonist on the development of adiposity, dysglycemia, and atherosclerosis in a mouse model.	Degarelix treated mice gained less visceral fat, had improved glucose tolerance, and had significantly smaller necrotic plaque areas compared with leuprolide and orchiectomized mice.
Knutsson A, et al. Sci Rep [35]	Male ApoE−/− mice A shear stress modifier was used to produce both advanced and more stable plaques in the carotid artery	*N* = 6–9/group To investigate the effects of degarelix and leuprolide on atherosclerotic plaques in high-fat-fed ApoE−/− mice.	Leuprolide treated mice had increased areas of necrosis observed in stable plaques and greater inflammation vs degarelix treated mice (demonstrated by greater macrophage accumulation within the plaques).
Chen et al. J. Clinical Endocrinology and Metabolism [36]	RT-PCR was used to analyze peripheral blood mononuclear cells (PBMCs)	To identify the presence of GnRH and GnRH receptors in human peripheral blood mononuclear cells	GnRH receptors are also expressed in human peripheral blood mononuclear cells. The endogenous production of GnRH by lymphocytes may act as an autocrine or paracrine factor to regulate immune functions.
Tanriverdi et al. 2005 clinical and experimental immunology [37]	Peripheral blood mononuclear cells (PBMCs) isolated from healthy males	*N* = 6 To investigate the inflammatory effects of GnRH-I and/or GnRH-II on human PMBC proliferation in males.	GnRH-I and GnRH-II receptors increased the expression of IL-2 mRNA in a dose-dependent manner which was associated with increased proliferation of PBMCs.

## Data Availability

The datasets generated during and/or analyzed during the current study are available in the PubMed repository.

## Supplementary Materials

Supplementary Materials Supplementary Figure 1: flowchart of the selection process. A comprehensive search in PubMed and ClinicalTrials.gov with the following key terms: “prostate cancer” AND “agonists” AND “antagonist” AND “cardiovascular” according to the recommendations in the PRISMA (Preferred Reporting Items for Systematic Review and Meta-analyses) statement. The initial search led to 106 publications, review articles, and editorials being removed, with priority given to relevant clinical trials, meta-analyses, and real-world evidence observational studies published in the last 10 years. In total, 29 articles were retrieved as a result of the selection process. Other articles included are related to preclinical studies, FSH review articles, and articles related to combination therapy with abiraterone and enzalutamide.
