# Supplementary material for: Assessment and Mitigation of Cardiovascular Risk for Prostate Cancer Patients: A Review of the Evidence
Source: Int J Clin Pract. 2022 May 17;2022:2976811. doi: 10.1155/2022/2976811 (PMC9158798; doi:10.1155/2022/2976811)
Supplement: Supplementary Materials — Supplementary Figure 1: flowchart of the selection process. A comprehensive search in PubMed and ClinicalTrials.gov with the following key terms: “prostate cancer” AND “agonists” AND “antagonist” AND “cardiovascular” according to the recommendations in the PRISMA (Preferred Reporting Items for Systematic Review and Meta-analyses) statement. The initial search led to 106 publications, review articles, and editorials being removed, with priority given to relevant clinical trials, meta-analyses, and real-world evidence observational studies published in the last 10 years. In total, 29 articles were retrieved as a result of the selection process. Other articles included are related to preclinical studies, FSH review articles, and articles related to combination therapy with abiraterone and enzalutamide. [file 2976811.f1.docx]

**10**

**6**

**articles**

**3**

**2**

**articles**

Articles

excluded

if

:

Review, Editoria

l

**29**

**articles**

D

uplicates removed

**5**

**RCT**

**’**

**s**

**9**

**meta**

**-**

**a**

**nalyses**

**9 RWE**

**6**

**O**

**ther**

**Supplementary Figure 1.** Flow Chart of the selection process. A comprehensive search in PubMed and clinicaltrials.gov with the following key terms; “prostate cancer” AND “agonists” AND “antagonist” AND “cardiovascular” according to the recommendations in the PRISMA (Preferred Reporting Items for Systematic Review and Meta-analyses) statement. The initial search led to 106 publications, review articles and editorials were removed, with priority given to relevant clinical trials, meta-analyses and real-world evidence observational studies published in the last 10 years. In total 29 articles were retrieved as a result of the selection process. Other articles included relate to pre-clinical studies, FSH review articles and articles related to combination therapy with abiraterone and enzalutamide.
